# Transmembrane Chemical Absorption Process for Recovering Ammonia as an Organic Fertilizer Using Citric Acid as the Trapping Solution

**DOI:** 10.3390/membranes14050102

**Published:** 2024-04-29

**Authors:** Ricardo Reyes Alva, Marius Mohr, Susanne Zibek

**Affiliations:** 1 Institute of Interfacial Process Engineering and Plasma Technology (IGVP), University of Stuttgart, Pfaffenwaldring 31, 70569 Stuttgart, Germany; ricardo.reyes-alva@igvp.uni-stuttgart.de; 2 Fraunhofer Institute for Interfacial Engineering and Biotechnology (IGB), Nobelstr. 12, 70569 Stuttgart, Germany

**Keywords:** transmembrane chemical absorption, TMCS, TMCA, membrane stripping, hydrophobic membrane contactor, ammonia recovery, supernatant, reject water, citric acid

## Abstract

Membrane contactors are among the available technologies that allow a reduction in the amount of ammoniacal nitrogen released into the environment through a process called transmembrane chemical absorption (TMCA). This process can be operated with different substances acting as trapping solutions; however, strong inorganic acids have been studied the most. The purpose of this study was to demonstrate, at laboratory scale, the performance of citric acid as a capturing solution in TMCA processes for recovering ammonia as an organic fertilizer from anaerobic digestor reject water using membrane contactors in a liquid–liquid configuration and to compare it with the most studied solution, sulfuric acid. The experiments were carried out at 22 °C and 40 °C and with a feed water pH of 10 and 10.5. When the system was operated at pH 10, the rates of recovered ammonia from the feed solution obtained with citric acid were 10.7–16.5 percentage points (pp) lower compared to sulfuric acid, and at pH 10.5, the difference decreased to 5–10 pp. Under all tested conditions, the water vapor transport in the system was lower when using citric acid as the trapping solution, and at pH 10 and 40 °C, it was 5.7 times lower. When estimating the operational costs for scaling up the system, citric acid appears to be a better option than sulfuric acid as a trapping solution, but in both cases, the process was not profitable under the studied conditions.

## 1. Introduction

The growing population, which currently stands at 8 billion inhabitants, and the economic development of the planet are crucially increasing the exploitation of natural resources while fires, droughts, and other consequences of climate change are simultaneously decreasing their availability [1,2]. If the sustainable development goal (SDG) of global food security by 2030 set by United Nations (UN) is to be met, around 750 million people who are currently undernourished should be able to improve their daily diet as soon as possible [3,4]. This will require a maintenance or increase in global efforts for sustainably producing food, which intrinsically encompasses a need for increasing fertilizer production.

Atmospheric N_2_ is hardly reactive, and thus, not readily available for use in fertilizers, providing a reason to consider the availability of nitrogen-containing fertilizers as a matter of concern. In order to break such a molecular bond, high amounts of energy are required; thus, in nature, it can only be metabolized by a few microorganisms [5]. Hence, the rate at which N-fixation occurs in nature is not enough to cover the global nitrogen requirements. Since the early 1900s, it has been possible to produce fertilizers at a much quicker pace thanks to the anthropogenic alternative for N-fixation, the Haber–Bosch process (HBP). It is estimated that around two thirds of global food is produced using fertilizers containing N-compounds originally obtained from ammoniacal-N synthesized through the HBP [6].

Although, the HBP represents an important positive milestone in science, industry, and agriculture, it has an important disadvantage, as it demands significant amounts of energy per amount of treated nitrogen due to the high temperature and pressure required. Another disadvantage is that the CH_4_ employed for the process mainly comes from fossil fuels, such as natural gas. These requirements result in an approximate energy use of 35E50 MJ∙kg^−1^ N [7]. Considering a production of around 199.4 Mt of fertilizer in 2018 and an increasing annual rate of 4% [8,9], the energy consumed by this process alone accounts for approximately 2% of the global energy consumption (178,899 TWh∙a^−1^) [10,11].

**Figure 1:**
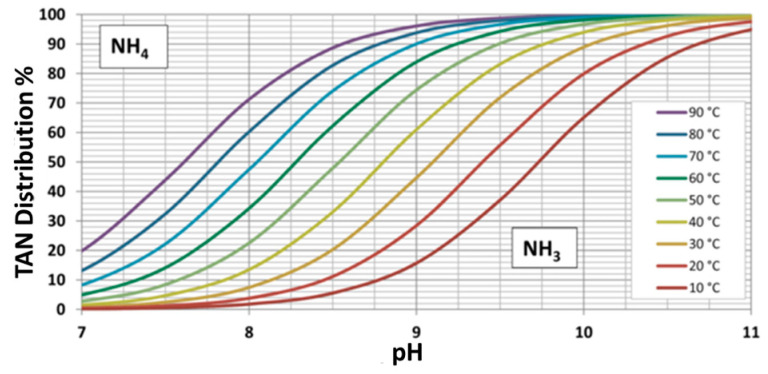
Ammonium/Ammonia equilibrium. Reprinted/adapted with permission from [16].

Given the aforementioned figures, it is clear that wastewater treatment plants (WWTPs) should undergo a transition from conventional disposal facilities towards wastewater biorefineries (WWBRs) for enabling the sustainable use of nitrogen. Given the fact that the stream in a WWTP with the highest concentration of total ammoniacal nitrogen (TAN) is the reject water after anaerobic digestion, the implementation of nitrogen recovery processes at this stage is more logical [12]. Ammoniacal nitrogen is the sum of dissociated ammonia (NH_4_^+^) and undissociated ammonia (NH_3_), and its chemical equilibrium is mainly affected by pH and to a lesser extent by temperature, as depicted in Figure 1 [13]. The construction of WWBRs would reduce the amount of valuable TAN released to the atmosphere in an unusable form while bringing nitrogen into a circular economic cycle. In Germany, the average contribution of total Kjeldahl nitrogen to a WWTP per person equivalent is 11 g per day [14]. Considering that in small and medium-sized WWTPs 10–30% of the influent nitrogen load ends up in the reject water [15], the TAN potentially available for recovery would be 1.1–3.3 g N∙population equivalent (PE)^−1^∙d^−1^.

Membrane contactors (MCs) are among the current technologies available that can be used for the recovery of nitrogen in wastewater. These special membranes are hydrophobic, allowing gaseous substances to permeate while liquid substances are retained. This feature is conveniently used for the selective recovery of gaseous ammonia by absorbing it into a trapping solution (TS), typically a strong acid, flowing on the permeate side of the membrane.

Multiple authors [17,18,19] have already pointed out that this process does not involve adsorption but rather an absorption step. Therefore, the proper name for this nutrient recovery procedure should be Transmembrane Chemical Absorption (TMCA) instead of the commonly seen term Transmembrane Chemisorption (TMCS). Other terms related to this same process include liquid–liquid membrane contactor (LLMC) [20], hydrophobic gas-permeable membrane [21], membrane stripping [22], and different combinations of these terms [17,23].

TMCA for ammonia recovery was first tested in the 1980s [24], and currently there are already a couple of full-scale facilities [25,26,27]. However, there are still challenges encompassing this process that hinder its expansion [17], including fouling, negative process economics, and low N-concentration products, among others.

While any substance with a pH lower than 9 can be used as a capturing solution, the most studied TMCA process uses sulfuric acid as a TS, obtaining ammonium sulfate as a product. On the one hand, this approach has the advantage of using a cheap acid and obtaining a well-established fertilizer [17]. On the other hand, the use of such a strong acid presents safety risks for the plant operators, and the inorganic product has a low market value (0.43 €∙kg^−1^) and is mainly suitable for agricultural fields with sulfur deficiency [28,29]. Moreover, the fertilizers obtained with the use of inorganic acids are, by definition, not useable for organic farming, hindering the expansion of ammonia recovery technologies. Therefore, the use of organic acids as trapping solutions should be further explored to allow the introduction of nutrient recovery technologies into organic farming practices.

Previous studies have tested different trapping solutions in the TMCA process, such as sulfuric acid [30,31], nitric acid [32,33], phosphoric acid [32,33], and deionized water [34,35], among others. Furthermore, there are studies on the efficiency of organic trapping solutions as well, such as sunflower oil [36]. To the best of the authors’ knowledge, there are only two studies on citric acid as an alternative organic capturing solution for the recovery of ammonia, in which the obtained product was ammonium citrate with a higher selling value than ammonium sulfate.

However, one of the related studies was performed during a conventional ammonia stripping–scrubbing process [37] and the other one in a TMCA set up to treat a synthetic solution where the membranes were suspended in air, resulting in a gas–liquid membrane contactor configuration [28]. This means that there is information lacking on the use of citric acid as a trapping solution for recovering nitrogen with membrane contactors in liquid–liquid configurations. There are also few studies on the economic performance of the TMCA process [12,38]. The number of studies that included an economic analysis is even lower for the systems that considered a trapping solution different to sulfuric acid [28].

The purpose of this study is to test, for the first time, the performance of citric acid as an organic trapping solution when treating anaerobic digestate reject water in a liquid–liquid membrane contactor setup and to reduce the knowledge gap on the economic performance of the process by providing an estimation of the operational expenses, including the use of auxiliary chemicals, for scaling up the system in a small WWTP with a treatment capacity for 25,000 PE.

## 2. Materials and Methods

### 2.1. Digestate and WWTP

The anaerobic digestion liquors, also known as reject water, process water, centrate, filtrate or supernatant, used for the experiments were collected from the municipal wastewater treatment plant in Erbach (Baden-Württemberg), Germany. The collection point was after the dewatering stage of the digestate carried out with a chamber filter press. The collected samples were refrigerated at 4 °C before carrying out the experiments.

Before the experimental setup was filled, the water was filtered with a laboratory-scale ultrafiltration module (Memos, Pfullingen, Germany) with a cut-off range of 100 kDa.

The operational expenses for scaling up the system were estimated considering the information provided by the operators of the WWTP in Erbach as well. This small plant has a design capacity of 25,000 population equivalent, and the produced reject water is around 35 m^3^∙d^−1^. The income obtained from selling the obtained products was calculated using the prices reported by previous studies without considering the achievable product concentration through TMCA. The complete set of parameters used for the economic calculations can be found in Table S3.

### 2.2. Reagents and Hydrophobic Gas-Permeable Membrane

Two different trapping solutions were used, sulfuric acid (H_2_SO_4_, CAS 7664-93-9, 37%) and citric acid (C_6_H_8_O_7_, CAS 5949-29-1, ≥99%) diluted with demineralized water to a weight concentration of 37%. For increasing the pH of the feed water to 10 and 10.5, a 30% sodium hydroxide solution was prepared using NaOH pellets (CAS 1310-73-2).

The membrane contactor used for all experiments consists of a cased module of hollow fiber membranes (3M^TM^ Liqui-Cel^TM^ EXF-2.5x8) made of polypropylene. Table 1 summarizes the features of this product relevant for this study.

### 2.3. Experimental Setup

As recommended by the supplier, the hollow fiber membrane contactor (HFMC) was mounted vertically for enabling a complete draining of the system supported with air supply. The configuration was set as a closed-loop circuit, in which the feed water flow was from bottom to top through the shell side of the module, while the acidic trapping solution was set to flow countercurrent from the feed water, from top to bottom, through the lumen side of the fiber membranes.

**Figure 2:**
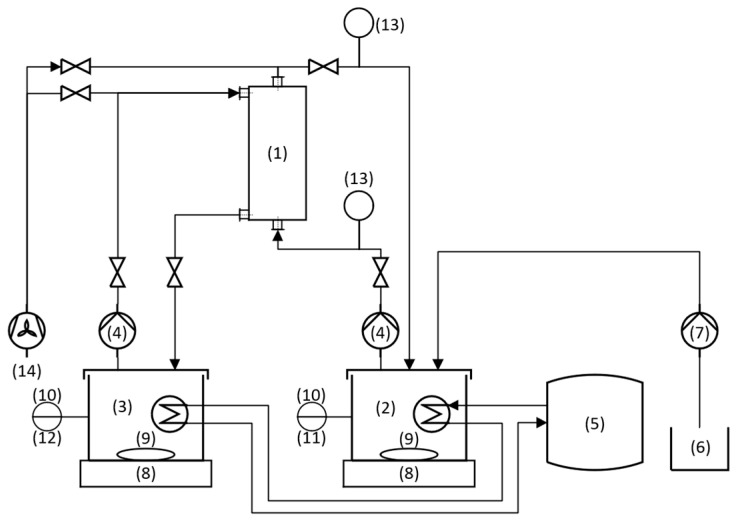
Closed-loop bench-scale setup (1) Hydrophobic membrane contactor; (2) Feed water tank; (3) Trapping solution tank; (4) Peristaltic pump; (5) Temperature controller; (6) NaOH container; (7) Dosing pump; (8) Bench scale; (9) Magnetic agitators; (10) pH sensor; (11) Temperature sensor; (12) Multimeter; (13) Barometer; (14) Air supply (for cleaning).

Both the feed water (1 L) and trapping solution (0.25 L) were stored in double-walled borosilicate cylindrical reactors. Both containers were placed over a bench-scale WS 6000-6 (BOSCHE, Damme, Germany) for measuring the weight change in the reactors during operation. Both substances were continuously stirred with magnetic agitators. Peristaltic pumps 520-U (Watson Marlow, Cornwall, UK) were used for safely pumping both substances throughout their respective circuits. A membrane pump Gamma/4 (ProMinent, Heidelberg, Germany) connected to a measuring and control unit Dulcometer D1cb (ProMinent, Germany) was set for dosing the NaOH solution whenever the pH dropped below the desired value. For each experiment, the initial and final volumes of NaOH were measured. Finally, a temperature controller F12 (Julabo, Seelbach, Germany) was connected to both reactors for ensuring a constant temperature throughout the system. The described setup is depicted in Figure 2.

Two sets of experiments were carried out: the first one served as a benchmark, using sulfuric acid as the trapping solution, and the second one using citric acid as an alternative trapping solution. A factorial experimental design was carried out for both sets, where the modified parameters were pH (10 and 10.5) and temperature (22 °C and 40 °C). All experiments at 40 °C were duplicated, and the results reported are the obtained averages with their respective standard deviation shown with error bars in all graphs. The experiments at 22 °C were performed one time, considering that there are related studies operating at a similar temperature [28]. Each run lasted 20 min from the moment the desired pH and temperature conditions were reached in the system.

### 2.4. Analytical Methods

The measured parameters for the feed water were pH, temperature, total nitrogen (TN), total ammoniacal nitrogen, total alkalinity, total suspended solids (TSSs), chemical oxygen demand (COD), and reactive orthophosphate (PO_4_-P). The parameters measured for the trapping solutions were pH, temperature, TN, and TAN.

The pH value and temperature of the feed water were measured with a PHER 112 SE sensor (ProMinent, Germany) and a PT100 sensor, respectively, connected to a Dulcometer D1cb controller unit (ProMinent, Germany). The pH of the trapping solutions was continuously measured with a portable multimeter WTW-Multi 3320 (Xylem, Atlanta, GA, USA). TN, TAN, and COD were measured following the persulfate digestion, salicylate, and dichromate methods, respectively, and with a spectrophotometer DR3900 (Hach, Ames, IA, USA). An important remark is that the acidic samples must be diluted and/or neutralized for the proper measuring of TN and TAN when applying the previously described analytical method. Total alkalinity was measured by titrating a H_2_SO_4_ 0.02 N solution into the samples until reaching a value of pH 4.5. The water vapor transport was calculated from the weight increase in the reactor holding the acidic trapping solution. Finally, the use of sodium hydroxide was measured by subtracting the final from the initial volume.

### 2.5. Data Analysis

The analysis of data was done with mass values of NH_3_-N, which will be referred to as NH_3_, instead of concentrations. This type of analysis was done considering that the initial volumes for the feed (1 L) and trapping solutions (0.25 L) were different. Even if both initial volumes were equal, the final volumes would be different due to the water vapor transport through the membrane. For the latter reason, total sample volumes were measured at the end of each experiment to obtain the actual nitrogen mass content. In summary, working with mass units instead of concentrations is more appropriate for TMCA studies where

(1) $$m_{{NH}_{3}}\left(mg\right)=C_{NH3}\left(\frac{mg}{L}\right)\times V\left(L\right)$$

Removed NH_3_ was obtained from the difference between the initial and final NH_3_ in the feed water.

(2) $$m_{NH3,\;rem}\left(mg\right)=m_{NH3\;F,o}\left(mg\right)-m_{NH3\;F,f}\left(mg\right)$$

where the subscripts *rem*, *F*, *o*, and *f*, refer to “removed”, “feed”, “initial”, and “final”, respectively.

Similarly, the removal rates were calculated with Equation (3).

(3) $$\;y_{NH3,rem.\;}\left(\%\right)=1-\left(\frac{m_{NH3\;F,f}\left(mg\right)}{m_{NH3\;F,o}\left(mg\right)}\right)\times 100$$

The recovered ammonia in the permeate (subscript *p*) was obtained by measuring the mass of NH_3_ in the trapping solution (subscript *TS*) at the end of the experiment.

(4) $$m_{NH3,p}\left(mg\right)=m_{NH3\;TS,\;f}\left(mg\right)$$

Consequently, the recovery rate is given by Equation (5)

(5) $$y_{NH3\;recovery}\left(\%\right)=\left(\frac{m_{NH3\;TS,f}\left(mg\right)}{m_{NH3\;F,o}\left(mg\right)}\right)\times 100$$

The NH_3_ losses in the system were calculated from the difference between the recovery and removal rates.

(6) $$y_{NH3\;losses}\left(\%\right)=y_{NH3\;rem,}\left(\%\right)-y_{NH3\;recovery}\left(\%\right)$$

Removed ammonia mass transfer *J_NH3, rem._* was calculated using Equation (7) following Podstawczyk et al. [39].

(7) $$J_{NH_{3,\;rem.}}\left(\frac{g}{m^{2}\cdot d}\right)=\frac{\left(c_{NH3\;F,o}-c_{NH3\;F,f}\right)\left(\frac{g}{L}\right)\cdot V_{F,avg.}\left(L\right)}{A_{m}\;\left(m^{2}\right)\cdot t\left(d\right)}$$

The recovered ammonia mass transfer *J_NH3, p_*_._ was obtained from Equation (8), according to Xu and He [40] and Reig et al. [32].

(8) $$J_{NH3,\;p.}\left(\frac{g}{m^{2}\cdot d}\right)=\frac{c_{NH3\;TS,f}\left(\frac{g}{L}\right)\cdot V_{TS,\;\;avg.}\left(L\right)}{A_{m}\;\left(m^{2}\right)\cdot t\left(d\right)}$$

Finally, Equation (9) was used for calculating the water vapor transport occuring during the experiments.

(9) $$j_{w}\left(\frac{L}{m^{2}\cdot h}\right)=\frac{V_{w}\left(L\right)}{A_{m}\left(m^{2}\right)\cdot t\left(h\right)}$$

where *V_W_* stands for the volume of water transported from the feed water tank into the trapping solution tank.

## 3. Results and Discussion

### 3.1. Feed Water Characterization

The supernatant collected from the WWTP in Erbach was analyzed before and after filtration and after carrying out the experiments. The filtration pretreatment served mainly for reducing the risk of clogging the membrane contactor, considering that the manufacturer’s recommendation is to operate with feed water with a total suspended solids content of less than 5 mg∙L^−1^. Another advantage of the pretreatment is to reduce the contents of organic compounds, which have been identified as the main sources of fouling in the TMCA process [22]. Table 2 gives a summary of the feed water characterization upon collection, after pretreatment, and the average values of all experiments.

Analyzing the efficiency of the ultrafiltration module used is not the purpose of this article. However, it is relevant to show the type of pretreatment undertaken and the characteristics of the filtered feed water used in the experiments for enabling comparability with other studies.

The alkalinity of the collected supernatant fell within the expected range (2000–4000 mg CaCO_3_∙L^−1^) for anaerobic digester liquors in domestic wastewater treatment plants [41]. After the experiments, the alkalinity increased up to two times the initial value due to the added NaOH for increasing the feed water’s pH. Regarding electrical conductivity, the filtered water still had more than four times the typical average value of water used for irrigation (900 µS∙cm^−1^) in agricultural fields, and the value reached 7910 µS∙cm^−1^ for the treated water, also due to the dosed sodium hydroxide [34]. Considering the relatively high values of pH, alkalinity, and conductivity, it is recommended to return this treated feed water back to the inlet of the wastewater treatment plant, as it could not be used directly for irrigation.

The TAN concentration of 483.5 mg∙L^−1^ falls within the typical range for this type of process water in this particular WWTP (400–700 mg∙L^−1^) [42]. It is important to notice that the filtered water complies with a ratio of TAN to inorganic carbon <1 (<0.21), identified by Daguerre-Martini et al. [43] as adequate for coupling a TMCA system with a CO_2_-stripping module for reducing costs for chemical use. Thus, it is worth considering such a configuration for future trials when treating the reject water from this same WWTP.

Another important characteristic of the filtered water is that its TN concentration was equal to its TAN concentration, in other words, 100% of the nitrogen content was ammoniacal nitrogen. Taking this relationship into consideration, total nitrogen was not measured in the subsequent stages of the study. Furthermore, the fact that there was no organic nitrogen present in the filtered water also means that proteins were completely removed, which is one of the main recommendations from Zarebska et al. [22], who characterized proteins as main foulants in membrane contactors used in TMCA processes. Nitrogen-free organic compounds were permeated through the UF-module, as noted from the COD value; however, the concentration was almost halved (403.7 mg∙L^−1^) with respect to the sampled reject water (744.7 mg∙L^−1^). The feed water’s concentration of ammoniacal nitrogen was estimated for each test, as its value decreased from the original 463 mg∙L^−1^ down to 430 mg∙L^−1^ over the experimental period, possibly due to volatilization. After the experiments, the TAN concentration decreased to less than 33 mg∙L^−1^, meaning that the removal rate was at least >92%, and this will be described in more detail in the next chapters.

Finally, the orthophosphate content was measured due to the risk it poses of clogging the system considering that at pH > 9 it tends to precipitate into different types of salts depending on the ions present in the water. Conveniently, the PO_4_-P content in the filtered water was relatively low (6.9 mg∙L^−1^) and was not expected to cause damage to the system. Besides, the membrane contactor was cleaned after every trial according to the manufacturer’s recommendation to prevent fouling [44]. At the end of the trials, the orthophosphate concentration decreased to a range of 1.38 to 3.67 mg∙L^−1^, meaning that P-salt precipitation took place in the system.

Finally, total suspended solids also pose an important threat to the system integrity, but the UF system was capable of reducing its content from 103.7 mg∙L^−1^ down to 15 mg∙L^−1^. Although this value is still higher than that recommended by the membrane manufacturer (<5 mg∙L^−1^) [45], the risk of clogging was reduced by flowing the feed water through the shell side of the module and not through the lumen side. Besides, other authors [22,26] found microfiltration technology to be sufficiently effective as pretreatment for a similar operation as the one in this study.

### 3.2. Performance Comparison between Citric Acid and Sulfuric Acid

#### 3.2.1. NH_3_ Mass Transfer and NaOH Consumption

Table 3 summarizes the results on ammonia mass transfer using Equations (7) and (8) for all experiments as well as the average base consumption for increasing the pH value of the feed water to 10 or 10.5. Further details on these results can be found in Tables S1 and S2. The fluxes give information on the speed at which ammonia can be recovered by the TMCA process under the studied conditions. Although the experiments lasted 20 min, the results were extrapolated linearly to present the achievable ammonia mass transfer in the system per day for allowing an easier comparability with other studies [28,46].

The first point to notice is that the difference in ammonia mass transfer when considering the removal values under the same pH and temperature conditions, and using Equation (7), is minimal. For instance, at pH 10 and 22 °C, the values for citric acid and sulfuric acid were 21.36 and 21.63 g∙m^−2^∙d^−1^, respectively, resulting in a 1.2% deviation, and at pH 10.5 and 40 °C, the results were 22.33 and 22.30 g∙m^−2^∙d^−1^, respectively. This is because the rate at which the ammoniacal nitrogen is being removed from the feed water is mainly determined by the operational pH and temperature, while the trapping solution plays a secondary role. Even if there was no membrane connected to the system, at high pH (and temperature) the ammonia gas would tend to leave the liquid phase. For this reason, it is more pertinent to consider the recovered mass for comparing ammonia mass transfer within the TMCA system.

When considering the extrapolated flux of recovered ammonia, it can be noticed that at pH 10, the results for sulfuric acid are higher than those for citric acid by approximately 1 g∙m^−2^∙d^−1^, at both temperatures. When operating a suspended TMCA system with an e-PTFE membrane at 25 °C and pH > 8, Soto-Herranz et al. [28] obtained values approximately four times higher than the ones in this study, where the average N recovered by the sulfuric acid was 24 g∙m^−2^∙d^−1^ and by the citric acid was 18 g∙m^−2^∙d^−1^. The higher ammonia flux obtained in that study could be a product of the higher feed N-concentration or the synthetic nature of the emitting solution. Similarly, in a pilot-scale study, Riaño et al. [47] recovered 14.48 g∙m^−2^∙d^−1^ from manure digestate at pH 9.11 and 25 °C when using sulfuric acid as the trapping solution. The main differences in this study with respect to the previously mentioned one were the high initial TAN concentration of >2700 mg∙L^−1^, the hydraulic retention time (7 days), and the membrane material (e-PTFE).

On the other hand, the recovered ammonia flux difference between sulfuric and citric acids was reduced when operating at pH 10.5. Here, the difference was only 0.28 g∙m^−2^∙d^−1^ at 22 °C and 0.59 g∙m^−2^∙d^−1^ at 40 °C, in both cases higher for sulfuric acid.

Regarding chemical consumption, naturally, the experiments in which the feed water was conditioned to have pH 10 required less NaOH than those where the pH was set to 10.5. For reaching pH 10.5, the mean alkali consumption increased by 27% compared to when conditioning the water to pH 10. The obtained values are lower than the ones obtained by Wäeger-Baumann and Fuchs [19], who experimented using anaerobic digester effluent and reported a consumption of 13 and 21 mL of NaOH 50% per liter of treated water for reaching pH values of 10 and 11, respectively. On the other hand, Richter et al. [48] reported for a full-scale plant an average NaOH 50% consumption of 4.05 mL∙L^−1^ for reaching a pH value of around 10.3 when treating reject water from a municipal WWTP. Furthermore, Ulbricht et al. [25] reported an average chemical consumption of 1 mL∙L^−1^ NaOH 50% for conditioning industrial wastewater to pH > 11 in a full-scale plant. The relatively low consumption of NaOH in the latter study is presumably given by the nature of the industrial wastewater, possibly with low alkalinity; however, that parameter was not reported by any of the cited studies, and thus, this hypothesis cannot be confirmed.

#### 3.2.2. Removal, Recovery, and Losses of NH_3_

**Figure 3:**
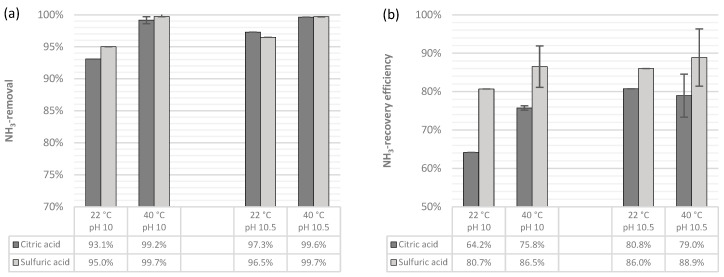
(**a**) NH_3_ removal efficiency in the TMCA process when using C_6_H_8_O_7_ vs. H_2_SO_4_ as the trapping solution (**b**) NH3 recovery efficiency comparison between citric and sulfuric acid in the TMCA process.

As can be noticed in Figure 3, the NH_3_ removal and recovery rates followed the expected behavior based on theory and previous experience [21,25,28]. The removal efficiency for both trapping solutions when operating at 40 °C was almost complete (>99%) and considerably higher than that at 22 °C. When the pH was set to 10 and the TS was sulfuric acid, it increased by 4.7 percentage points (pp), and for citric acid, the removal was 6.1 pp higher. Similarly, at pH 10.5 and with a temperature increase from 22 °C to 40 °C, the removal of ammonia increased 3.2 and 2.3 pp when using sulfuric acid and citric acid, respectively.

Interestingly, the removal efficiency was always slightly higher when using sulfuric acid than when using citric acid under similar operating conditions, except for the case when the pH was set to 10.5 and the temperature to 22 °C. For this setup, there was a 96.5% removal of NH_3_-N when using H_2_SO_4_ and 97.3% removal when using C_6_H_8_O_7_. However, at 25 °C, Soto-Herranz et al. [28] reported 12% higher removal rates for sulfuric acid compared to citric acid; thus, the outlying result in this study can be ignored until further tests are performed.

The removal efficiency at both pH values and at 22 °C (>90%) was significantly higher than the one reported by Soto-Herranz et al. [28], who, when operating a suspended TMCA system at 25 °C and a pH > 8 for treating a synthetic solution, reported an overall removal efficiency with citric acid of 34%. The higher removal rate shown in this study could be mainly due to the higher operational pH of the feed water. Figure 1 shows that under the experimental conditions of the previously cited study, around 30% of the ammoniacal nitrogen was in the form of free ammonia, which is practically the same percentage of nitrogen recovered in the previously cited study.

The recovery rates followed the same trend, where the sulfuric acid showed a higher capacity for absorbing ammonia than did citric acid. At pH 10 and 22 °C, the inorganic acid was able to recover 80.7% of the NH_3_ present in the feed water, while the citric acid’s recovery efficiency was 16.5 pp lower. When increasing the temperature to 40 °C and keeping the pH at 10, the efficiency of the trapping solutions increased 5.8 and 11.6 pp for sulfuric acid and citric acid, respectively. This increase in temperature reduced the efficiency gap between sulfuric acid and citric acid as trapping solutions from a difference of 16.5 down to 10.7 pp. In a full-scale plant, treating anaerobic digester centrate and using the same type of membrane contactor and a higher concentration of sulfuric acid for the capturing solution (78%), Richter et al. [26] obtained 85% recovery when operating at pH 11, which is similar to the performance seen in this study.

On the other hand, there was little or no improvement in TAN recovery when changing the temperature from 22 °C to 40 °C at a fixed pH of 10.5 for both TSs. This can be explained by looking at Figure 1, in which it can be noticed that at pH 10.5 and 20 °C, around 94% of TAN was present as free ammonia, and increasing the temperature to 40 °C only increased the ammonia gas fraction to approximately 97%. At pH 10.5 and 22 °C, sulfuric acid recovered almost the same percentage of TAN (86%) as when operating at pH 10 and 40 °C (86.5%). However, keeping the pH at 10.5 but increasing the temperature to 40 °C increased the efficiency by only 2.9 percentage points, whereas at pH 10.5 and at both temperatures, the performance of citric acid improved 5 extra pp compared to the setup with pH 10 and T = 40 °C. Considering that in these results, increasing the temperature did not increase the mean recovery efficiency when using citric acid as a capturing solution, it appears that this extra effort for conditioning the feed water is not recommended. Another fact worth noticing from these results is that, at pH 10.5 and 22 °C, the recovery efficiency gap between H_2_SO_4_ and C_6_H_8_O_7_ was the lowest, with only 5.2 pp. Thus, when operating under these conditions, there seems to be a small technical advantage of using sulfuric acid over citric acid as a TS.

At both pH values and at 22 °C, the recovery rates obtained by the citric acid (64.2% and 80.8%) were also higher than the one reported by Soto-Herranz et al. [28], who obtained an overall recovery efficiency with citric acid of 22.1%. The ammonia stripping–scrubbing system with citric acid of Jamaludin et al. [37] was set to 70 °C with a feed water pH of 9.07, resulting in a recovery efficiency of 90.12% after 240 min. The differences between these reported results are rational considering that the three systems were significantly different in terms of the type, pH, and temperature of the feed water, system setup, trapping solution concentration, and experimental time.

**Figure 4:**
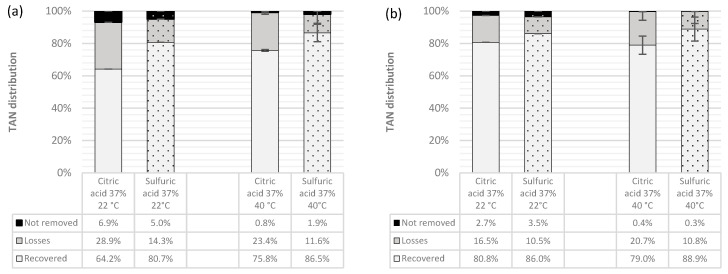
TMCA process efficiency comparison when using citric and sulfuric acids as trapping solutions (**a**) at pH 10 (**b**) at pH 10.5.

Similarly, Figure 4 offers a complete overview of the average removed (recovered + lost) TAN and also of the non-removed NH_3_-N throughout the experiments. With these graphs it is possible to show that in all cases, most of the removed TAN was actually recovered and not just simply blown out of the system through volatilization [46]. However, it is also worth noticing that while non-removed TAN was rather constant for both TSs under the same operating conditions, there were higher NH_3_-N losses in the system when using citric acid than when using sulfuric acid.

Assuming that the losses at the feed water tank remained constant in all experiments under the same operating conditions regardless of the type of acid used, the difference in the percentage of lost ammonia must occur in the capturing solution tank. Seen from another perspective, if the mass transfer resistance on the acid side was negligible for both acids as has been proved for the case of H_2_SO_4_ [17], the difference between the removal and recovery rates should be similar for both trapping solutions if all parameters in the system remain equal. The fact that this condition was not met suggests that the mass transfer resistance on the acid side was not negligible for the case of citric acid. Thus, contrary to when using sulfuric acid as a trapping solution, when using citric acid, the rate at which the permeated NH_3_ reacts with the protons on the gas–liquid interface is not instantaneous. This can be due to the fact that citric acid is a weak triprotic acid, whose dissociation in solution is not complete, making less protons available to react with the permeated ammonia [49].

#### 3.2.3. Water Vapor Transport

**Figure 5:**
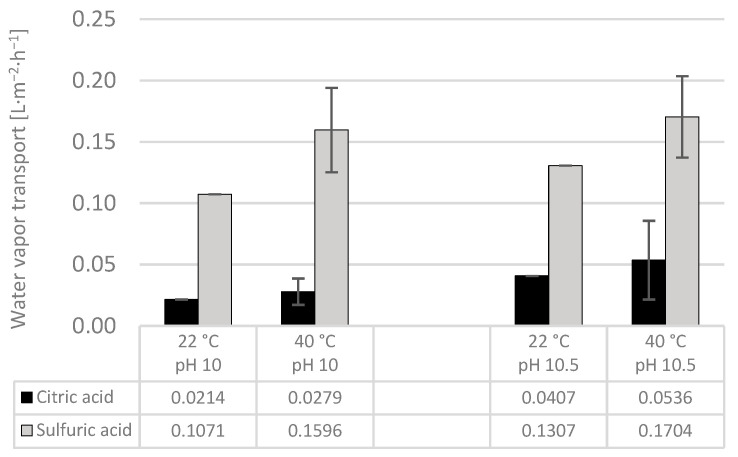
Water vapor transport in TMCA using C_6_H_8_O_7_ and H_2_SO_4_ as trapping solutions.

Figure 5 shows the results obtained for water vapor transport (WPT) throughout the experiments, which refers to the water permeating to the trapping solution tank. This is an unwanted phenomenon in the TMCA process because the higher the amount of water transported, the higher the dilution of the obtained product. The diffusion of water vapor through the membrane is given by its partial pressure difference along the shell and lumen sides [17].

It is clear that in this category, citric acid performs better than sulfuric acid in the sense that it absorbed less water under all studied conditions, and thus, the product became less diluted. The obtained results could be explained from the fact that sulfuric acid is known as a highly hygroscopic substance, meaning that it has a high capacity to absorb humidity [50].

For both capturing solutions, the WPT was lower at 22 °C, as expected, where after 20 min and at pH 10, the citric acid tank absorbed 0.02 L∙m^−2^∙h^−1^ and the sulfuric acid around five times more water. Keeping the pH at 10 but increasing the temperature to 40 °C had a noticeable impact on water vapor transport when using sulfuric acid, with a 49% increase, while for citric acid, it only increased 30%.

Furthermore, when keeping the temperature at 22 °C but increasing the pH from 10 to 10.5, the WPT for citric acid almost doubled, and for sulfuric acid it increased 22%. Finally, at a constant temperature of 40 °C but changing the pH from 10 to 10.5, the water vapor transport for citric and sulfuric acids increased 92% and6.7%, respectively.

Similarly, Vecino et al. [20] obtained slightly lower water vapor transport results (0.015 L∙m^−2^∙h^−1^) when operating the exact same model of membrane contactor for recovering ammonia from an eluent (1.7 g N∙L^−1^) from a sorption process, at 22 °C and pH 12, and using nitric acid as the trapping solution. On the other hand, when operating a full-scale plant (440 m^2^) with sulfuric acid as the TS, treating industrial wastewater, Ulbricht et al. [25] reported 119 and 150 L∙h^−1^, translated to 0.27 and 0.34 L∙m^−2^∙h^−1^ when conditioning the feed water to pH 11, at 30 °C and 40 °C, respectively. The differences with respect to this study might be due to the following:

- The higher operational pH, where Vecino et al. operated their system at pH 12 and Ulbricht et al. [25] at pH 11;
- The difference in the water flow rate, where Vecino et al. operated at 450 mL∙min^−1^;
- The temperature differences between the feed and acid tanks, as none of the cited authors included a heating system for the acid tank;
- Or the different characteristics of the capturing solutions.

### 3.3. Economic Estimations

The performance of the trapping solutions could also be considered in terms of the resultant capital (CAPEX) and operational expenses (OPEX), which can be the main parameter evaluated when deciding whether to use this process. Table 4 provides a summary of a few parameters considered for performing the cost estimation for the hypothetical construction of a TMCA plant at a WWTP (25,000 PE) similar to the one where the reject water was collected (Erbach, Germany), following the same configuration proposed for this study and considering the results obtained at bench scale. The operational parameters of the feed water for performing the cost estimation were set to pH 10 and a temperature of 40 °C. These operational settings were chosen taking as reference the work of Noriega-Hevia et al. [12], who identified these conditions as the most efficient in monetary terms for a similar process configuration. However, it is important to take into account that this type of conclusion is specific for the conditions considered by the authors, including the country (Spain), reject water inflow (600 m^3^∙d^−1^), and year of analysis (2021).

It is clear that for scaling up the studied system, it would not be possible to use the same models of pumps and membranes as the ones used in the laboratory. For the purpose of increasing the accuracy of the analysis, the technical data of equipment that has been assessed as adequate for scaling up the process were considered. However, the purchasing costs for such devices can vary significantly from supplier to supplier; thus, the capital expenses were not included in our calculations. Only the energy requirements for the circulation pumps were considered and not for the chemical dosing pumps, which are negligible when analyzing the complete process [12]. The thermal energy costs were also not listed, considering an ideal case in which the requirements can be fully met by using the energy obtained from the biogas produced by the anaerobic digestor on-site. The results are presented as a function of the amount of ammoniacal nitrogen to be treated at the WWTP to allow comparability with other studies.

The monetary savings from reducing the amount of nitrogen returned to the inflow line of the WWTP are not included in these calculations. However, the environmental impact can be estimated considering that nitrous oxide (N_2_O) has a global warming potential of 265 kg CO_2_-eq∙kg^−1^, [54,55] and that N_2_O emissions represent approximately 4% of the nitrogen load [12]. As described in the introduction of this study, the estimated amount of potentially recoverable nitrogen was 1.1 to 3.3 g N∙PE^−1^∙d^−1^_._ This amount of nitrogen that will not be contributing to the WWTP influent represents a potential emission reduction of 0.044 to 0.132 g N_2_O∙PE^−1^∙d^−1^, translated to approximately 106.4–319.2 t CO_2_∙a^−1^. Furthermore, a reduction in the nitrogen load entering a WWTP would also imply a reduction of around 10% in energy consumption related to the aeration in the nitrification process [12,55].

With the current (2024) energy, fertilizer, and chemical prices in Germany and the recovery rates presented in this study, scaling up the process at a small WWTP does not appear to be profitable with any of the two studied trapping solutions. When using both acids, the costs for energy and chemical use overpass the revenue obtained from selling the obtained product. In the case of sulfuric acid, the overall losses per day were calculated as 17.4 € per kilogram of treated TAN, and for the case of citric acid, the losses were 14.9 €∙d^−1^∙kg N^−1^.

The total costs when using sulfuric acid were estimated as 18.9 €∙d^−1^∙kg N^−1^, while for citric acid, the value increased to 24.7 €∙d^−1^∙kg N^−1^. This difference is due to the fact that when using citric acid, double the amount of acid is required for recovering the same amount of nitrogen according to Jamaludin et al. [37] and Soto-Herranz et al. [28]. Adding to that, the price of this organic acid (1.06 €∙kg^−1^) is more than double that of sulfuric acid (0.48 €∙kg^−1^). However, even when the associated costs are higher when operating the TMCA process with citric acid, the overall process economics are better due to the higher revenue obtained from selling ammonium citrate instead of ammonium sulfate, considering the ammonium citrate prices reported by Soto-Herranz et al. [28]. In the absence of commercially available prices for ammonium citrate solutions, it is recommended to contact farmers near the site of operation to inquire the price that they would be willing to pay for this product at the obtained concentrations.

In both cases, the use of NaOH represents the highest share in the operational expenses, being 74% and 57% for sulfuric and citric acid, respectively. Taking this fact into consideration, if such a plant were to be constructed, it would be highly advisable to consider a CO_2_-stripping unit to reduce or avoid the consumption of NaOH and therefore, its associated costs. The pH of swine manure can be increased 1 unit in one hour, reaching a value of approximately 8.5 solely through aeration [21]. García-González et al. [21] reported a 57% reduction in OPEX when including aeration in their ammonia recovery process from swine manure due to the reduction of NaOH requirements.

Another way of improving the process economics would be by increasing the selling price of the obtained product. In the projected scenario, in order to reach a breakeven point in which the expenses equal the profit from selling the products, it would be necessary to increase the price of the ammonium sulfate to 5.6 € per kilogram, which would be 13 times higher than the current price (0.43 €). In the case of the citric acid, the price would need to be increased to 2.7 € per kilogram to achieve the breakeven point, which is 2.5 times higher than the current selling price (1.06 €).

Furthermore, the pretreatment with the UF module also represents up to 13% of the OPEX without considering maintenance costs. While the UF module proved to be efficient for reducing the risk of fouling in the system, microfiltration (MF) has also been identified as sufficient for the same purpose [26,45]. Besides, the membrane supplier’s recommendation is to have a suspended solids content of no more than 5 mg∙L^−1^, which should be achievable even without membrane technology. Substituting the UF system for a less energy-intensive technology would have an impact not only on the operation but also, and more importantly, on the capital expenses associated with the proposed process.

Drastically modifying just one parameter for making the proposed plant economically attractive is neither realistic nor convenient. A more feasible approach would be to make modifications to multiple parameters. That strategy would include the previously mentioned proposals: modifying the pretreatment to a less energy-intensive system, e.g., sand filtration, implementing a CO_2_-stripping module for reducing NaOH consumption, reaching a long-term agreement with chemical suppliers for obtaining better stock prices, and increasing the selling price of the obtained product. The latter could be more difficult to achieve because the selling price is ruled by the market. The farmers that could be interested in using the obtained fertilizers would certainly refuse to pay a higher price than that offered by other suppliers. Here, political action would be needed to support the commercialization of sustainable and/or organic fertilizers coming from this type of green technology. Potential political support could be in the form of subsidies for their environmental impact achieved by the implementation of TMCA plants, such as the reduction in CO_2-_eq_._ emissions from the reduction in energy requirements for the aeration process and from the reduced N_2_O emissions.

## 4. Conclusions

This study aimed to determine if citric acid could function as a trapping solution in TMCA processes with a similar technical and economic efficiency as the one obtained by sulfuric acid. According to the results obtained at laboratory scale under defined operational conditions, the ammonia recovery efficiency of the process is slightly lower with citric acid than with sulfuric acid. However, the unwanted water vapor transport through the membrane is lower when using citric acid, making the obtained product less diluted and producing ammonium citrate, which is marketable as an organic fertilizer in contrast to ammonium sulfate. Finally, the overall operational expenses are lower with sulfuric acid. Nevertheless, the revenue obtained from the organic product when using citric acid is higher, making the process more profitable in comparison to when using sulfuric acid. The operational expenses have to be reduced in order to make TMCA a fully sustainable process. For having a better notion of the performance of citric acid as a trapping solution in full-scale facilities, future studies testing it at pilot and/or large scales would be required. In this context, the most important research gaps include the long-term operation of TMCA systems using citric acid as a capturing solution, an achievable nitrogen concentration of the final product, water vapor transport reduction, and concentration techniques.

## Figures and Tables

**Table 1:** Membrane contactor characteristics.

Feature	Unit	Value
Membrane type	-	Hollow fibers X-50
Material	-	Polypropylene
Effective surface area	m^2^	1.4
Area density	m^2^∙m^−3^	1871
Number of fibers	-	11,000
Pore size	µm	0.04
Fiber outer diameter	mm	0.3
Fiber inner diameter	mm	0.2
Cartridge outer diameter	mm	66.6
Cartridge inner diameter	mm	6.4
Packing grade	-	0.25

**Table 2:** Characteristics of feed water used in the experiments.

Parameter	Units	Reject Water	UF-Filtered Water	After TMCA *
pH	-	7.8	7.8	10–10.5
Electrical conductivity (σ)	µS∙cm^−1^	4787.5	4040.0	4210–7910
Total alkalinity (TA)	mg CaCO_3_∙L^−1^	2343.0	2201.0	2520–4510
Total suspended solids (TSS)	mg∙L^−1^	103.75	15	**
Total nitrogen (TN)	mg∙L^−1^	516	430–463	***
Total ammoniacal nitrogen (TAN)	mg∙L^−1^	483.5	430–463	<1–32.4
Chemical oxygen demand (COD)	mg∙L^−1^	744.0	403.7	*
Orthophosphate (PO_4_-P)	mg∙L^−1^	26.9	6.9	1.38–3.67

* Summarized results from all experiments ** = Not measured *** = Same as TAN.

**Table 3:** Ammonia mass transfer and chemical consumption in the studied TMCA system configuration using sulfuric and citric acids as capturing solutions under different operational conditions.

	Feed Water					J_NH3, rem._	J_NH3,p._	NaOH (30%)
TS	pH	Temp.	TAN_o_ * [mg]	TAN_f_ * [mg]	TAN_p_ * [mg]	Flux * [g∙m^−2^∙d^−1^]	Flux * [g∙m^−2^∙d^−1^]	Addition [mL∙L^−1^]
C_6_H_8_O_7_	10	22 °C	447.8	30.9	287.3	21.36	4.02	8.01 ± 1.34
		40 °C	441	3.7	334.1	22.48	4.67	
H_2_SO_4_	10	22 °C	445.4	11.7	359.4	21.63	5.02	
		40 °C	437	1.5	403.8	22.41	5.64	
C_6_H_8_O_7_	10.5	22 °C	435	22.2	351.3	21.74	4.91	10.19 ± 1.1
		40 °C	435.8	1.1	344	22.33	4.81	
H_2_SO_4_	10.5	22 °C	430.2	15.2	371.5	21.29	5.19	
		40 °C	435	1.3	386.3	22.30	5.4	

TS = trapping solution; o = initial; f = final; p = permeate; rem. = removal; * = average values.

**Table 4:** Summary of the parameters considered for our process analysis.

		H_2_SO_4_	C_6_H_8_O_7_
Parameter	Unit	Value (% of total costs)	
WWTP Erbach			
TAN load in reject water [42]	kg∙d^−1^	21	
Pretreatment costs			
Energy [51]	€∙d^−1^∙kg N^−1^	2.5 (13%)	2.5 (10%)
TMCA costs			
Energy	€∙d^−1^∙kg N^−1^	0.7 (4%)	0.7 (3%)
NaOH [52]	€∙d^−1^∙kg N^−1^	14 (74%)	14 (57%)
Trapping solution [28,53]	€∙d^−1^∙kg N^−1^	1.7 (9%)	7.6 (30%)
Total Costs	€∙d^−1^∙kg N^−1^	18.9	24.7
Sales and profit			
Fertilizer production	kg∙d^−1^	70.5	195.7
Product price [28,29]	€∙kg^−1^	0.43	1.06
Revenue	€∙d^−1^∙kg N^−1^	1.4	9.9
Profit/Losses	€∙d^−1^∙kg N^−1^	−17.4	−14.9
Product price for breakeven point	€∙kg^−1^	5.6	2.7

## Data Availability

The data presented in this study are available on request from the corresponding author. The data are not publicly available due to a confidentiality agreement among the involved institutions.

## Supplementary Materials

The following supporting information can be downloaded at: https://www.mdpi.com/article/10.3390/membranes14050102/s1, Table S1: Ammonia distribution in the studied TMCA system configuration using sulfuric and citric acid as capturing solutions under different operational conditions. Table S2:Ammonia mass transfer in the studied TMCA system configuration using sulfuric and citric acid as capturing solutions under different operational conditions. Table S3: Operational expenses estimations.
