# Supplementary material for: Transmembrane Chemical Absorption Process for Recovering Ammonia as an Organic Fertilizer Using Citric Acid as the Trapping Solution
Source: Membranes (Basel). 2024 Apr 29;14(5):102. doi: 10.3390/membranes14050102 (PMC11123178; doi:10.3390/membranes14050102)
Supplement: Supplementary file 1 [file membranes-14-00102-s001.zip › membranes-2940662-supplementary.pdf]

Transmembrane Chemical Absorption Process for Recovering Ammonia as an Organic Fertilizer Using Citric Acid as the Trapping Solution (Supplementary Information)

Ricardo Reyes Alva, Marius Mohr, and Susanne Zibek

Table S1. Ammonia distribution in the studied TMCA system configuration using sulfuric and citric acid as capturing solutions under different operational conditions

TS	pH	T	TAN_o [mg]	Avg. TAN_o [mg]	TAN_f [mg]	Avg. TAN_f	TAN_p [mg]	Avg. Tan_p [mg]
$C_6H_8O_7$	10	22 °C	447,8	447,8	30,9	30,9	287,3	287,3
		40 °C	441,4	441	6,1	3,7	336,9	334,1
			441		1,2		331,2	
H_2SO_4	10	22 °C	445,4	445,4	11,7	11,7	359,4	359,4
		40 °C	437,4	437	1,5	1,5	427,7	403,8
			436,6		1,5		379,9	
$C_6H_8O_7$	10,5	22 °C	435,0	435	22,2	22,2	351,3	351,3
		40 °C	436,6	435,8	0,8	1,1	320,3	344,0
			435,0		1,3		367,7	
H_2SO_4	10,5	22 °C	430,2	430,2	15,1	15,1	371,4	371,5
		40 °C	439,8	435	1,4	1,3	358,1	386,3
			430,2		1,0		414,4	

Table S2. Ammonia mass transfer in the studied TMCA system configuration using sulfuric and citric acid as capturing solutions under different operational conditions

	pH	T	$J_{NH3, rem.}$ [$g \cdot m^{-2} \cdot d^{-1}$]	Avg. $J_{NH3,rem.}$ [$g \cdot m^{-2} \cdot d^{-1}$]	$J_{NH3, p}$ [$g \cdot m^{-2} \cdot d^{-1}$]	Avg. $J_{NH3,p}$ [$g \cdot m^{-2} \cdot d^{-1}$]	NaOH [mL]	Avg. NaOH [mL]
$C_6H_8O_7$	10	22 °C	21,4	21,4	4,0	4,0	6,4	8,01
		40 °C	22,4	22,5	4,7	4,6	7,2	
			22,6		4,6		7,0	
H_2SO_4	10	22 °C	21,6	21,6	5,0	5,0	8,0	
		40 °C	22,4	22,4	5,3	5,2	9,2	
			22,4		5,2		10,3	
$C_6H_8O_7$	10,5	22 °C	21,7	21,7	4,9	4,9	9,2	10,19
		40 °C	22,4	22,3	4,4	4,8	9,3	
			22,3		5,1		9,7	
H_2SO_4	10,5	22 °C	21,3	21,3	5,1	5,1	12,2	
		40 °C	22,5	22,3	5,0	5,3	11,3	
			22,1		5,7		9,5	

Table S3. Operational expenses estimations

Pretreatment				
**Parameter**	**Unit**	**Value**	**Value per kg of TAN**	**Reference**
Membrane				
Membrane flux	$L \cdot m^{-2} \cdot h^{-1}$	25		
Membrane area	m^2	60	2,9	
Pump				
Head*	bar	5		
Nominal power	kW	8,25		
Operational level	% full capacity	80		
Operational time	$h \cdot d^{-1}$	24		
Pump energy consumption	$kWh \cdot d^{-1}$	158,4	7,5	
Energy price	$€ \cdot kWh^{-1}$	0,3306		[1]
**Energy cost**	**$€ \cdot d^{-1}$**	**52,4**	**2,5**	
**TMCA**				
**Parameter**	**Unit**	**Value**	**Value per kg of N**	**Reference**
Circulation pump				
Nominal power	kW	2,2		
Operational level	% full capacity	85		
Operational time	$h \cdot d^{-1}$	24		
Pump energy consumption	$kWh \cdot d^{-1}$	44,88	2,14	
Energy price	$€ \cdot kWh^{-1}$	0,3306		[1]
**Energy cost**	**$€ \cdot d^{-1}$**	**14,8**	**0,7**	
Chemical consumption				
NaOH 30% price	$€ \cdot kg^{-1}$	0,77		[2]
NaOH 30% density	$kg \cdot L^{-1}$	1,36		
NaOH 30% requirement	$L \cdot m^{-3}$	8,01	0,38	
	$kg \cdot m^{-3}$	10,9	0,52	
	$kg \cdot d^{-1}$	381,3	18,16	
**NaOH cost**	**$€ \cdot d^{-1}$**	**293,6**	**14,0**	
H_2SO_4 50% price	$€ \cdot kg^{-1}$	0,48		[3]
H_2SO_4 50% consumption	$H_2SO_4:TAN_r$	3,5		[4]
	$kg \cdot d^{-1}$	73,5		
**H_2SO_4 cost**	**$€ \cdot d^{-1}$**	**35,3**	**1,7**	
$C_6H_8O_7$ 50% price	$€ \cdot kg^{-1}$	1,08		[5]
$C_6H_8O_7$ 50% consumption	$kg \cdot d^{-1}$	147	7	[5]
**$C_6H_8O_7$ cost**	**$€ \cdot d^{-1}$**	**158,8**	**7,6**	

Sales				
H_2SO_4				
Recovery efficiency	%	86,5		
Recovered TAN	$kg·d^{-1}$	18,2		
Ammonium sulfate produced	$kg·d^{-1}$	70,5		
Ammonium sulfate price	$€·kg^{-1}$	0,43		[6]
**H_2SO_4 sales**	**$€·d^{-1}$**	**30,3**	**1,4**	
$C_6H_8O_7$				
Recovery efficiency	%	75,8		
Recovered TAN	$kg·d^{-1}$	15,9		
Ammonium citrate production	$kg·d^{-1}$	195,70		
$C_6H_{11}NO_7$ price	$€·kg^{-1}$	1,06		[5]
**$C_6H_{11}NO_7$ sales**	**$€·d^{-1}$**	**207,4**	**9,9**	

References

[1] Statista, Strompreise für industrie und Gewerbe seit 2006, Available online at: https://de.statista.com/statistik/daten/studie/154902/umfrage/strompreise-fuer-industrie-und-gewerbe-seit-2006/

[2] Chemieshop 24, Natronlauge 30%, Available online at: https://www.chemieshop24.de/natronlauge-30-en896-1200kg-container/1002796701000

[3] Chemieshop 24, Schwefelsäure 50%, Available online at: https://www.chemieshop24.de/schwefelsaeure-50-en899-1300kg-container/1000419201004

[4] Noriega-Hevia, G.; Serralta, J.; Seco, A.; Ferrer, J. Economic analysis of the scale-up and implantation of a hollow fibre membrane contactor plant for nitrogen recovery in a full-scale wastewater treatment plant. *Separation and Purification Technology* **2021**, *275*, 119128, doi:10.1016/j.seppur.2021.119128.

[5] Soto-Herranz et al. Evaluation of Different Capture Solutions for Ammonia Recovery in Suspended Gas Permeable Membrane Systems. *Membranes (Basel)* **2022**, *12*, doi:10.3390/membranes12060572.

[6] Chemanalyst, Ammonium Sulphate Price Trend and Forecast, Available online at: https://www.chemanalyst.com/Pricing-data/ammonium-sulphate-64#:~:text=In%20Q1%202023%2C%20the%20Ammonium,and%20a%20bearish%20demand%20outlook.
